# Chicago Dental Society

**Published:** 1885-01

**Authors:** A. E. Baldwin


### CHICAGO DENTAL SOCIETY.

### DECEMBER MEETING.

REPORTED FOR THE INDEPENDENT PRACTITIONER BY A. E. BALDWIN, M. D.

The paper of the evening was by Dr. E. S. Talbot, and the subject was "*Contraria Contrariis Curantur*, as Applied to Dentistry." He said:

It was with surprise, not unmixed with dismay, that I received notice from the programme committee that a paper on the above subject would be expected from me. Just why I should have been chosen to discuss a dogma as fallacious as it is ancient, and which, in common with all dogmas of a medical character, I believe to be unworthy the support of liberal and enlightened minds, I cannot comprehend. Nevertheless, the fact remains, and the ancient mummy confronts me in very material form.

The subject of *Contraria Contrariis* is one which can only be handled by one of my medical opinion in a very gingerly fashion. More especially as there is little to do, save to build men of straw only to demolish them. If the committee have assumed that in assigning me this subject they are simply allowing the other side of the question an opportunity to be heard, as contrasted with the essay immediately preceding mine, they have made a mistake, and unwittingly done me an injustice.

It therefore becomes necessary for me to define my position by denying the implied impeachment, and disclaiming all faith in the so-called law of "*Contrariis*" as applied to medicine or dentistry It is none the less incumbent upon me to say something on this topic, for, though ancient and decrepit, it is as worthy our consideration as "*Similia Similibus Curantur*," or any other of the dogmatic beliefs entertained by medical sects.

This doctrine, as applied to medicine, was first taught by the Parisian school, of which Johannes Fernelius was the leader, in the sixteenth century.

Fernelius, in his famous work on Therapeutics, asserted that every disease must be combated by contrary remedies, for a remedy is that which drives out the disease. Now, that which drives acts violently; that which uses violence is in opposition; therefore, the remedy is always opposed to the disease, and no healing can take place except in virtue of the law of "*Contrariis.*"

It would be hard to imagine an assumption so replete with sophistry as this, and yet Fernelius was considered one of the great men of his time, and undoubtedly modified the medical literature of that period more than any of his co-laborers in science. But he was not content with the mere promulgation of a pet theory. He appreciated the fact that no position could be long sustained which was not susceptible of proof. To a man of the mental fertility and ingenuity of Fernelius, proofs for ever so fallacious a doctrine were not wanting. In the development of his law he includes under the term *Contrariis* not alone things possessing antagonistic elementary qualities, such as heat and cold, dry and wet, but all things which in any way differ, whether as to bulk or capacity, position, conformation, etc. Thus the compact and the soft, the thick and the thin, large and small, purity and impurity, are severally indicated by Fernelius as contraries, Such is the absurdity of the conclusion formed by a mind of superior attainments, when laboring under the incubus of a delusive dogma.

Modern science admits as contraries only those qualities of matter or forces which neutralize each other, which are also classed as contraries by Fernelius, as heat and cold, wet and dry, thus making his position still more untenable where these properties are contrasted with those mentioned further on in his classification, the north and the south winds, the centripetal and centrifugal forces.

The application of the doctrine of contraries, as advanced by Fernelius, to therapeutics, was most nonsensical, the precept being to treat diseases by remedies which differ from the disease itself, or its causes. To illustrate; we might brush the teeth with charcoal to prevent their decay, because charcoal differed from dental caries. So, even were the law of contraries correct, the practical precept is unworthy serious attention.

The law of contraries, as taught by Fernelius, is the law which the disciples of Hahnemann have for years been vainly endeavoring to father upon scientific medicine. Upon the absurdity of the alleged allopathic doctrine, they have endeavored to base the supremacy of a diametrically opposed dogma. What a waste of energy is this demolition of the allopathic man of straw!

Modern medicine is not based upon a peculiar dogma of any sort, and it surely is a great waste of thunder for the advocates of the law of similars to exhume the venerable mummy, and abuse him. There are fresh victims enough to allow the poor old fellow to repose in peace, even when the ancient masters of medical art, Hippocrates and Galen, recommend the use of remedies contrary to disease. They implied, not the mistaken views subsequently held by Fernelius, but that certain diseases were curable by remedies whose virtues were in direct antagonism to the cause or principle of the disease; in short, they believed in specifics. Not so very illogical, after all, for are we not at the present day, no matter what our creed, searching for specifics? Is it not true that the medical millennium will never arrive until we have our twenty-five thousand diseases, and our innumerable remedies, classified in as simple a formula as castor oil and constipation, peroxide of hydrogen and alveolar abscess? I wonder if my homeopathic brethren ever realize the fact that when they are searching for specifics they are bowing the knee to the mighty Hippocrates, and that the law of contraries was but one of the principles involved in his most liberal of all therapeutical propositions, viz., that diseases are cured sometimes by contraries, sometimes by similars, and sometimes by remedies the action of which is neither contrary nor similar, but which operate in an inexplicable manner. This same wise doctrine will apply to our dental specialty quite as aptly as to general medicine, and it is, in fact, the basis of modern medical and dental therapeutics. Nothing could be more liberal or comprehensive. Verily, Hippocrates has not been styled the father of medicine for naught, and he is quite likely to maintain his paternal supremacy for ages to come, in spite of comma baccili and the bacterial development of future generations.

Humanity receives one of its greatest boons in the breaking up of the general practice of medicine into specifics. To practice a specialty with success is glory enough for one man, and success is assured when the limits of inquiry and investigation are circumscribed by specific practice.

It is this, also, which has done much in reconciling the different schools of medicine. In the investigation of disease the school is forgotten, and the physician wanders into the realm of contraries and similars, or into theories which are neither of these. Hence those practicing the same specialty, but from different schools, consult frequently. Fortunately for our specialty, no other law is acknowledged save that laid down by the great Hippocrates. According to the law of contraries, the practitioner applies remedies regardless of cause or effect, without a definite knowledge of the case in the start. With the foundation of the present day in anatomy, physiology and chemistry, we are enabled by experience in both clinical observation and practice, to classify disease and to simplify remedies.

It is necessary in successfully practicing dentistry to be familiar with the anatomy of the parts to be operated upon, and the functions of these parts when in health. We can then appreciate the pathological conditions whenever they may present themselves. It is said by teachers of medicine that, having ascertained the cause of the disease, the remedy naturally suggests itself. This theory gains in practicability, and new discoveries in the nature and cause of disease are constantly being made. In the application of remedies, a thorough knowledge of their properties and therapeutic effect will aid greatly in the successful treatment of disease.

An old pactitioner of dentistry in this city has applied one simple remedy to many pathological conditions of the mouth for twenty years. He was ignorant of the cause of the disease he was treating, and of the effect of the drug used. He knew the drug to be harmless, and so freed his mind of the possibility of injurious effects. That he has not been a success is not surprising.

If by the application of the law of contraries to dentistry we accept Fernelius' conclusions, I should do so against all previous convictions, for, as before hinted, I am not an advocate of scientific dogmas, and, least of all, *Contraria Contrariis Curantur*, as applied to dentistry. If we look upon the application of contraries as understood by Hippocrates and Galen, where specifics stood in place of contraries, we may then apply this treatment intelligently in the practice of dentistry. A specific, according to Webster, "is a remedy which exerts a specific action in the prevention and cure of a disease." This definition would not accord with Fernelius' theory in the treatment of disease.

Dentistry has the advantage over other specialties in the ability to readily diagnose most lesions of the mouth, and to apply the remedies necessary. The first specific which deserves our attention is cleanliness. Next to godliness comes this specific, when applied to the teeth. Faces and hands are cleaned frequently in the day, but it requires the greatest effort to clean the teeth one-half that number of times.

Patients insist that they take the greatest care and cleanse the mouth very frequently, but appearances indicate either that is not properly done, or that the unpleasantness of this duty makes them exaggerate the frequency.

Dentists examine and fill teeth and dismiss patients without definite instructions as to the care of the teeth afterward. In a year they are ready for treatment again, with new approximal cavties, or a decay around old fillings. The gums are in an inflamed condition, saliva vitiated, and in a strong alkaline condition, and when we tell them this condition is caused by want of cleanliness, they assure us that they brush the teeth two or three times a day. Still the fact remains: dentists should not only clean the teeth of patients, but with proper brushes, string and quill, go through the programme of thoroughly instructing patients in the first specific in dental pathology.

The dentist himself should have his teeth in a condition to invite inspection, and illustrate the rules he lays down to his patients. In treating neuralgia it is plain that no dogma or pathy can be associated, for we cannot say this or that is a cure for neuralgia, but by removing the cause we radically cure the disease. The idea of applying the same remedy to a disease in all cases should be discouraged, for while a remedy may produce the desired effect in a majority of cases having the same disease, it may fail in some. This is particularly noticeable in the application of arsenic for the destruction of pulps, and in the application of medicaments for the obtunding of sensitive dentine, and of iodine and aconite in pericementitis, carbolic acid and peroxide of hydrogen in alveolar abscesses.

The dentist finds that nine-tenths of his time is occupied in filling cavities in decayed teeth. Does it occur to him that he treats these lesions from a homeopathic principle, viz., by treating the symptoms?

We examine the teeth and find the cavity (the symptom); we fill it with gold, tin, amalgam or oxy-phosphate, regardless of the cause of the decay in the teeth. Few teeth are filled between the ages of twelve and twenty that do not have to be refilled in five or ten years. Decay of teeth is on the increase, and will continue to be until the cause is treated, instead of the symptoms.

